# *SETBP1* dysregulation in congenital disorders and myeloid neoplasms

**DOI:** 10.18632/oncotarget.17231

**Published:** 2017-04-19

**Authors:** Nicoletta Coccaro, Giuseppina Tota, Antonella Zagaria, Luisa Anelli, Giorgina Specchia, Francesco Albano

**Affiliations:** ^1^ Department of Emergency and Organ Transplantation (D.E.T.O.), Hematology Section, University of Bari, Bari, Italy

**Keywords:** SETBP1, mutation, oncogene, Schinzel–Giedion syndrome, myeloid neoplasms

## Abstract

Myeloid malignancies are characterized by an extreme molecular heterogeneity, and many efforts have been made in the past decades to clarify the mechanisms underlying their pathogenesis.

In this scenario *SET binding protein 1* (*SETBP1)* has attracted a lot of interest as a new oncogene and potential marker, in addition to its involvement in the Schinzel-Giedon syndrome (SGS). Our review starts with the analysis of the structural characteristics of *SETBP1*, and extends to its corresponding physiological and pathological functions. Next, we describe the prevalence of *SETBP1* mutations in congenital diseases and in hematologic malignancies, exploring how its alterations might contribute to tumor development and provoke clinical effects. Finally, we consider to understand how *SETBP1* activation could be exploited in molecular medicine to enhance the cure rate.

## INTRODUCTION

In the past decade important progress has been made in understanding the extreme molecular heterogeneity characterizing myeloid neoplasms. Four years ago a new player appeared in this extensive landscape of molecular alterations. As often happens when a new gene involved in tumorigenesis is discovered, also in the case of *SET binding protein 1* (*SETBP1)* gene, the first studies reported its involvement in the pathogenesis of a congenital disorder, called Schinzel-Giedion syndrome (SGS). Depending on the type of mutation, the same gene may provoke different pathologic consequences; it is even more evident when the mutation hits at germline or somatic level.

In hematologic neoplasms, the discovery of *SETBP1* as a new oncogene has helped to better define the molecular characteristics of pathologies such as atypical Chronic Myeloid Leukemia (aCML), a disease initially defined only by negative characteristics, like the absence of BCR-ABL1 fusion. *SETBP1* mutations are found with different frequencies in almost all classes of myeloid disorders; these differences in the mutation prevalence highlighted the existence of a biological difference even between entities that in some cases have overlapping diagnostic criteria, as aCML and Chronic Myelomonocytic Leukemia (CMML).

Indeed, the described association of *SETBP1* mutations with a poor clinical outcome is an important beginning on which to build future studies to device therapeutic targeting.

In this review we will discuss the domains and functions of SETBP1 in normal biology and in pathologic contexts. In the last part, we will focus on how *SETBP1* alterations can be exploited in molecular medicine to enhance the cure rate.

## FROM GENE TO PROTEIN

The human *SETBP1* gene, originally called *SEB*, is located at the cytogenetic band q12.3 of chromosome 18, a region that contains candidate tumor suppressor genes associated with deletions in cancer and leukemia [1]. There are two isoforms of the *SETBP1* gene: the first (isoform a) encompasses a genomic region of 387613 pb, and the 6 exons encode an 9899 nt transcript, with a predicted protein of 1596 aa; the second one (isoform b) encompasses a genomic region of 197242 pb, and 4 exons encode a 1804 nt transcript with a predicted protein of 242 aa. Isoform b shares with isoform a the first 3 exons (UCSC; http://genome.ucsc.edu; release Dec 2013). Piazza et al. only observed the longest isoform expression by RNA sequencing and transcriptome profiling experiments in 13 aCML cases [2]. There is no other information about the expression, translation, localization and function of the shorter isoform.

**Figure 1:**
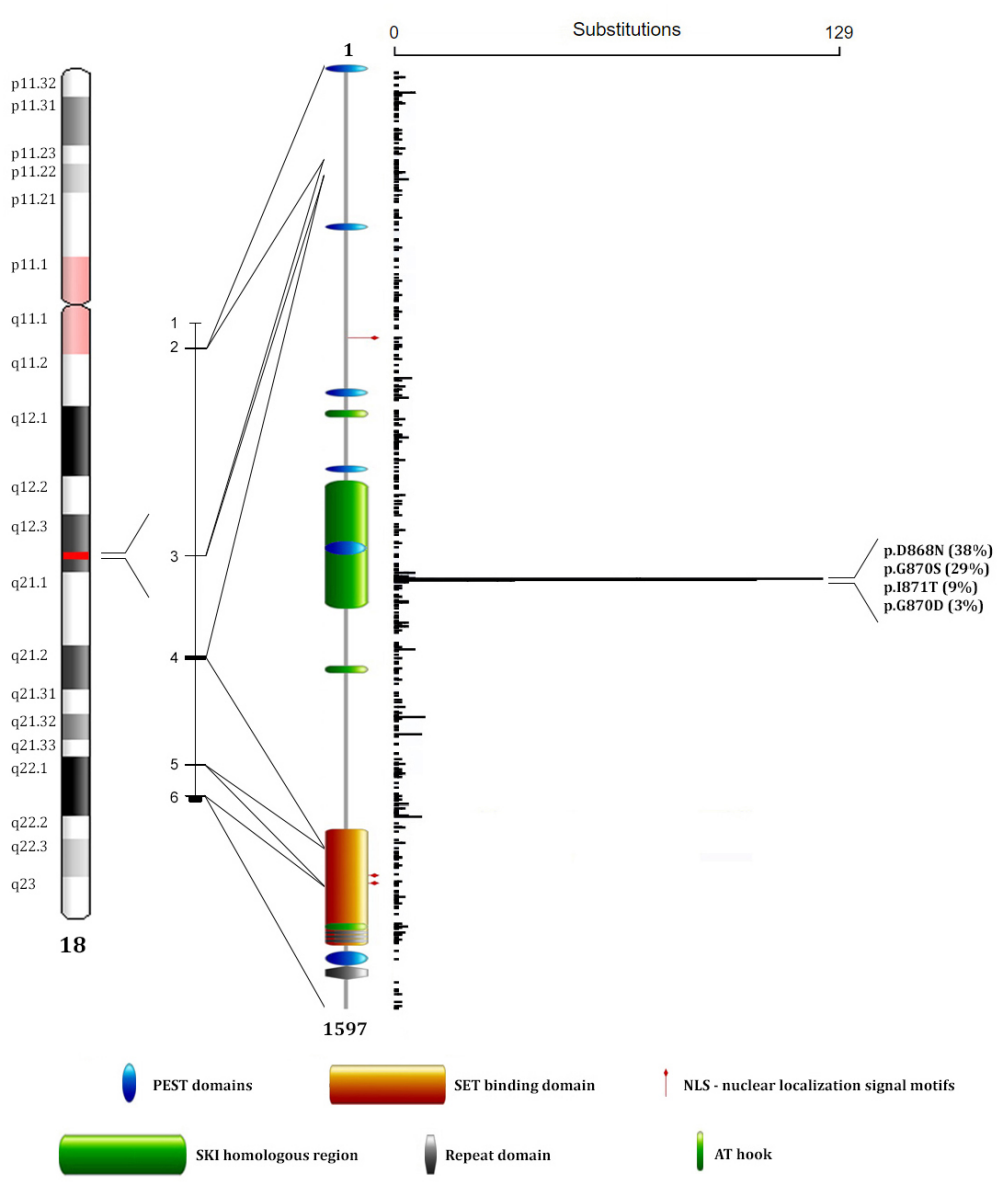
SETBP1 protein structure and mutations distribution From left to right: position map of the *SETBP1* locus on chromosome 18, *SETBP1* gene organization (isoform a), SETBP1 protein domains, and distribution of mutations reported on the COSMIC database (release Nov 2016). The image shows three AT hook domains (amino acids 584–596, 1016–1028, 1451–1463) [2], a SKI homologous region (amino acids 706–917) [2], a SET-binding domain (amino acids 1292–1488) [2], four repeat domains (amino acids 1466-1473, 1474-1481, 1482-1489, 1520–1543) [1, 2], three bipartite NLS motifs (amino acids 462-477, 1370-1384, 1383-1399) [1], and six PEST sequences (amino acids 1-13, 269-280, 548-561, 678-689, 806-830, 1502-1526) [1].

The SETBP1 protein, with an estimated molecular mass of 170 kDa [1], is composed of a SET-binding region, an oncoprotein SKI homologous region, three bipartite NLS (nuclear localization signal) motifs, three AT hook domains, six PEST sequences, three sequential proline-rich repeats, four KxKHKxK, eight LSxxL and ten PxxPS repeated sentences [1] (Figure 1).

The human SET-binding region of SETBP1 has a high identity with mouse Setbp1, suggesting that it may be conserved and that *SETBP1* may play an essential role in cells [1]. The binding of SETBP1 to the SET protein was identified by co-immunoprecipitation and DNA transfection experiments. It is well known that *SET* is a proto-oncogene that has a histone acetylation inhibitory activity and acts by inhibiting tumor suppressors as NM23-H1 and PP2A [3]. The cleavage of SET by Granzyme A during cytotoxic T lymphocyte-induced apoptosis removes the inhibition of NM23-H1, which translocates into the nucleus and cuts DNA [4, 5]. PP2A, a major protein phosphatase, can be bound and inhibited by SET and likely by a homeobox protein, HOX11, acting on several cell processes [6–10], such as cell proliferation, differentiation, and transformation [11, 12]. The effect of PP2A inhibition, observed in a human T-cell line, is the disruption of a G2/M cell-cycle checkpoint. SETBP1 regulates the SET inhibitory activity of PP2A and SET/PP2A interaction by its specific SET-binding [1]. Indeed, SETBP1 is a major counterpart of SET, and SET/SETBP1 interaction is stronger than that of SET/PP2A, in fact SETBP1 replaces PP2A in the SET/PP2A complex [1].

The SKI-homologous region of SETBP1 is so named because of the homology to the proto-oncogene SKI. SKI intervenes as a transcriptional co-repressor, inhibiting the transcription of target genes downstream of the Transforming Growth Factor-β (TGF-β) superfamily [13]. This region in SETBP1 could be involved in the regulation of the SKI/SKI homodimer and the SKI/SNON heterodimer, causing cellular transformation [14].

Three putative bipartite NLS motifs might be involved in signal-dependent nuclear transport of this protein across the nuclear pore [1].

The AT-hook motifs probably confer a DNA-binding capability to SETBP1; especially when present in multiple copies, AT-hook motifs can cause a DNA bending which could be crucial for transcriptional regulation [15]. Several proteins containing these motifs are components of chromatin remodeling complexes in yeast, Drosophila, and mammalian cells [16–18]. Through its AT-hook motifs, SETBP1 may control gene transcription as part of a chromatin-remodeling complex; this is also consistent with its predominantly nuclear localization [19]. Furthermore, other DNA sequence-specific transcription factors presumably act in recruiting SETBP1 to its target promoters, as AT-hook motifs do not recognize a specific DNA sequence [20].

## MECHANISMS OF ALTERATION OF SETBP1 FUNCTION

**Figure 2:**
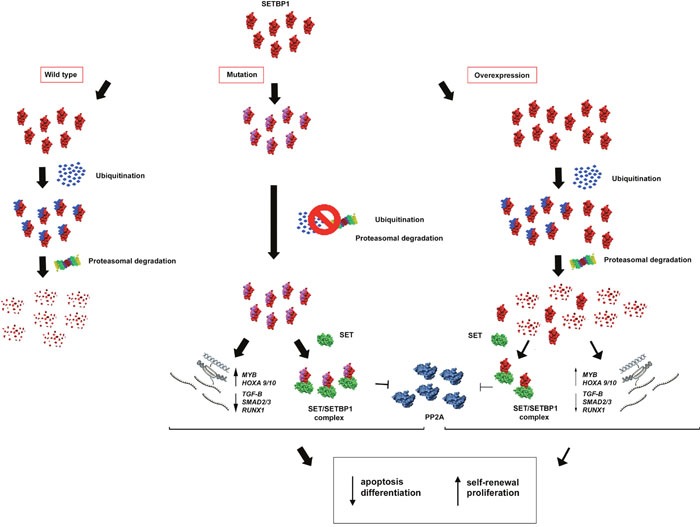
Hypothetical effects of SETBP1 alterations *SETBP1* gene mutations avoid ubiquitin binding causing a greater stability of the SETBP1 protein. In absence of protein degradation, SETBP1 binds SET and the SETBP1-SET complex inhibits PP2A. Moreover, through its activity as a transcription regulator, SETBP1 acts on the expression level of the *MYB, HOXA*, *RUNX1* genes and some targets of the *TGF-β* signaling pathway, activating a stem cell signature that includes apoptosis, differentiation and self-renewal. Milder effects are observed in the case of SETBP1 overexpression.

Various mechanisms can affect SETBP1 function. An altered expression was firstly observed in a translocation involving the *SETBP1* locus. Cristóbal et al. described in a patient with acute myeloid leukemia (AML) a novel t(12;18)(p13;q12) involving *ETV6*, resulting in overexpression of *SETBP1*, located close to the breakpoint [21]. The authors suggested that *SETBP1* overexpression protects SET from protease cleavage, increasing the amount of SET protein and leading to the formation of a SETBP1-SET-PP2A complex; this mechanism results in PP2A inhibition and proliferation of leukemic cells [21] (Figure 2). Besides, SETBP1 binds SET domains involved in the methylation of lysine residues on histone tails [22], and this binding could have important effects on both the inhibitory activity of SET, and on the Granzyme A mediated caspase-independent apoptosis induced by cytotoxic T lymphocytes; this could be a novel defense mechanism in leukemic cells [21]. In 2012 we reported *SETBP1* overexpression in a Primary Myelofibrosis (PMF) case with t(12;18)(p13;q12) evolving to AML. The observation of the concomitant downregulation of the intronic regulatory *MIR_4319* suggested a possible mechanism for *SETBP1* altered expression [23].

In 2010, Hoischen et al. described for the first time germline mutations in *SETBP1* in a congenital disorder called SGS [22, 24]. Then in 2013, Piazza et al. performed exome sequencing of eight aCML cases, identifying somatic *SETBP1* mutations in two cases. Subsequent analysis of the *SETBP1* mutation status of a further 70 aCMLs, 574 diverse hematological malignancies and 344 cancer cell lines revealed mutations in 24% cases. These analyses found a hotspot mutation region between codons 858–871; most SETBP1 mutations (92%) were the same as those seen in SGS, and were associated with higher white blood cell counts and a worse prognosis [2]. The *SETBP1* region where mutations cluster is highly conserved among vertebrates, and this suggests that it might have an important but still unknown biological role. According to the Eukaryotic Linear Motif (ELM) server, this region is a virtually perfect degron, i.e. an amino acids specific sequence that channels a protein to the initial degradation step. This degron in SETBP1 contains a consensus-binding region (DpSGXXpS/pT, where pS and pT are phosphorylated residues) for β-TrCP1, the substrate recognition subunit of the E3 ubiquitin ligase, and might be critical for protein degradation through ubiquitin binding [2]. When mutated, the SETBP1 protein is incapable of binding this E3 ligase subunit; this triggers a possible SETBP1 protein stability difference, in turn affecting the stabilization of SET. The stabilized SET protein can alter histone acetylation, or SET may directly bind and inhibit PP2A [2] (Figure 2).

Lastly, the *SETBP1* locus may be exposed to germline focal deletion. Filges et al. identified two patients with de novo chromosomal microdeletions in 18q12.3 featuring only *SETBP1*. *SETBP1* haploinsufficiency was suggested to be pathogenic but the phenotype seemed to be distinct from that of SGS [25] with milder developmental anomalies [26].

## *SETBP1* IN CONGENITAL DISEASE

Until 2010, the Schinzel–Giedion syndrome (SGS, MIM#269150) was presumed to be a monogenic condition, and remained undetermined if the heritability pattern was recessive or de novo dominant. The discovery of heterozygous mutations in the *SETBP1* gene by Hoischen et al. elucidated SGS inheritance as de novo dominant autosomal. To date 26 molecularly confirmed cases have been reported [22, 27–36].

The multisystemic involvement observed in SGS is explained by the observation that SETBP1 is ubiquitously expressed through the body [37]. In 2008, Lehman et al. suggested clinical criteria for the diagnosis, based on the co-occurrence of developmental retardation [38, 39] with a typical facial phenotype combined with hydronephrosis or typical skeletal malformations including a sclerotic skull base, wide occipital synchondrosis, increased cortical density or thickness, and broad ribs [40]. Sometimes features such as visual impairment, hearing loss [41], brain anomalies [42], neurological degeneration [43], and an increased incidence of embryonal tumors [44] have also been described. The presence of progressive developmental retardation and multiple malformations makes the disease extremely severe and the prognosis very poor. Patients usually die at an early age; the longest documented survival was 15 years [28].

Regarding the high incidence of malignancy in SGS, to date 9 cases of malignant tumors in SGS have been reported [31, 44–50]. It remains to be clarified whether these patients can tolerate therapy with cytotoxic agents and irradiation, as they show an increased tendency to infection. In any case the overall survival (OS) in patients with SGS and malignant tumors is poor [31]. As early diagnosis of SGS and early detection of malignancy might result in a better chance of survival, physicians should be aware of the high risk of malignancies in these patients.

Unlike from point mutations, *SETBP1* microdeletion seems to be associated to a different phenotype from SGS. The “*SETBP1* deletion phenotype” partially overlaps with the already described del(18)(q12.2q21.1) syndrome, featuring mild dysmorphic characters, mental retardation, impairment of expressive language and behavioral problems [25, 51]. Frequently the described deletions involve chromosomal 18 regions of various size, even if the minimum deleted region always contains the *SETBP1* locus; all the reported microdeletions are de novo and heterozygous [25, 52–54]. At molecular level, SETBP1 haploinsufficiency results in reduced expression, highlighting the observation that the “*SETBP1* deletion phenotype” is allele dose sensitive [25].

These data support the concept that different type of mutations in the same gene may provoke phenotypic variability as SETBP1 mutations causing SGS may generate a gain-of-function or a dominant-negative effect, whereas haploinsufficiency or loss-of-function mutations produce a milder phenotype [25].

## *SETBP1* IN CANCER

The suggestion of *SETBP1* involvement in leukemogenesis and tumorigenesis was firstly advanced in 2001 when Minakuchi et al. described its discovery [1]. The observation that SETBP1 specifically interacted with SET supported the hypothesis of a tumorigenic role, as a few years earlier, *SET* had been shown to be fused to *NUP214 (CAN)* in a case of acute undifferentiated leukemia [55]. Later, *SETBP1* was also described to be involved in a gene fusion, with *NUP98*, in a case of pediatric acute T cell lymphoblastic leukemia with t(11;18)(p15;q12) [56].

The first study involving a large cohort of cancer patients was conducted in 2010 by Cristòbal et al. which analyzed *SETBP1* expression level in 192 AML cases, finding an overexpression in 53 patients (27.6%). *SETBP1* overexpression was found to be associated with an unfavorable cytogenetic prognostic group featuring monosomy 7, and *EVI1* gene overexpression; *SETBP1*-overexpressed patients had a significantly shorter OS, and, when the patients were older than 60 years, the prognosis was very poor [21].

Several studies endeavored to shed light on the mechanisms by which SETBP1 exerts its oncogenic role. The effects of overexpression were studied by Oakley et al., who identified *SETBP1* as a novel regulator of leukemic stem cells (LSC) self-renewal in myeloid leukemias. They showed that *in vitro SETBP1* overexpression could efficiently immortalize myeloid progenitors and sustain self-renewal; in mice, *SETBP1* cooperated with *BCR-ABL1* in transforming committed myeloid progenitors, that normally lack a self-renewal capability, into LSCs, causing the development of myeloid blast crisis of chronic myeloid leukemia (CML). *SETBP1* overexpression was also observed in some CML advanced phase/blast crisis patients [15] in which PP2A activity was shown to be inhibited maybe through an increased SET expression induced by BCR-ABL1 [57].

Moreover, Oakley et al. were the first to find a novel transcriptional mechanism by which SETBP1 contributes to leukemia transformation via activating the *HOXA9* and *HOXA10* genes. *HOXA9* and *HOXA10* transcription levels in *SETBP1*-immortalized cells remained stable when cells were treated with 1,9-dideoxy-forskolin, a PP2A activator, raising the hypothesis that their activation could be independent of PP2A inhibition induced by SETBP1. In mouse and human myeloid progenitors the induced expression of the SETBP1 mutated form (either p.Asp868Asn or p.Ile871Thr) plays a role in immortalizing the cells by triggering the *HOXA* genes upregulation [58]. Indeed, the SETBP1 mutant form seemed to show a significantly more efficient colony formation capability and induce faster proliferation than the wild type counterpart [58, 59]. However, this oncogenic activity appears to be *HOXA*-genes-dependent, as silencing of either gene led to the loss of the proliferative ability both in the case of the *SETBP1* mutation and in the case of overexpression [15, 58].

Functional experiments on the SETBP1 p.Gly870Ser mutant, the second most frequent alteration in cancer, showed a significantly reduced PP2A activity as well as a greater PP2A phosphorylation at position Tyr307, a well-known marker of PP2A inactivation. Cells expressing SETBP1 p.Gly870Ser also had a higher proliferation rate compared to cells expressing wild-type SETBP1 [2]. Indeed, in the presence of SETBP1 p.Gly870Ser the expression of *LYN*, a *SRC* family kinase transcriptionally inhibited by PP2A, and of *PTGS2*, was higher, both in aCML cases and in transfected TF1 cells [11]. Because SETBP1 is a predominantly nuclear protein, whereas PP2A is also located inside the cytoplasm, additional unknown mechanisms are probably operative in this setting [2].

The poor protein degradation observed in the case of SETBP1 p.Gly870Ser mutation can be considered functionally equivalent to SETBP1 overexpression [2]. As most *SETBP1* mutations localize in the same region, a similar mechanism of action seems plausible also for them; the same conclusions were demonstrated also for p.Asp868Asn, the most frequent mutation [60]. Most mutations fall in the exon 4, outside the SET interacting domain, so they do not hit the DNA binding domains.

However, Makishima et al. observed some secondary AML (sAML) cases both with and without *SETBP1* mutations that showed high levels of wild-type mRNA. They hypothesized that the mechanisms through which the mutant SETBP1 protein exerts its oncogenic activity may be more complicated, and could involve an aberrant hypomethylation of the *SETBP1* promoter or alterations of upstream regulators such as *MECOM* [58, 61, 62] or of miRNAs such as *MIR_4319*, an intronic miRNA that was found to be downregulated in a case of PMF evolving to AML and expressing higher levels of *SETBP1* mRNA [23].

The finding of a strong association of *SETBP1* mutations with mutations in genes involved in pathways previously associated with a dismal prognosis lays the foundation for understanding the processes implicated in the malignancy pathogenesis and evolution. For example, it is known that PP2A can regulate the RAS-MAPK pathway via dephosphorylation of several substrates [63]; this interplay could explain the presence of *SETBP1* mutations in the pathogenesis of Juvenile Myelomonocytic Leukemia (JMML), which is mainly believed to be a *RAS* driven disease.

Furthermore, in an interesting study by Inoue et al. the relationship between mutations of *SETBP1* and *ASXL1* was explored, starting from the observation that in Myelodysplastic Syndrome (MDS) the high rate of co-occurrence of mutations resulted in a shorter OS and a higher incidence of leukemic transformation, and that the acquisition of the *SETBP1* mutation in *ASXL1*-mutated MDS occurs during disease progression [60, 64]. In the work by Inoue, the hypothesis that the *SETBP1* mutation confers a selective advantage and plays a role in disease evolution was demonstrated in a series of *in vitro* experiments in which the expression of SETBP1 p.Asp868Asn was shown to enhance myeloid colony formation of ASXL1-mutated cells, and to increase the *ASXL1* mutation-induced differentiation block of 32Dcl3 cells and primary bone marrow (BM) cells [60]. The increased stability gained by the mutated SETBP1 protein seems most likely to be a gain-of-function mutation, as overexpression of wild type SETBP1 exhibited milder effects than SETBP1 p.Asp868Asn.

Inoue et al. proposed, for the first time, an *in vivo* MDS-progressing-to-AML model expressing *ASXL1* and *SETBP1* mutations. In three independent experiments, mice transplanted with BM cells expressing both ASXL1-mutated and SETBP1 p.Asp868Asn developed AML and died, showing severe hepatosplenomegaly after a short latency; on the other hand the mice transplanted with BM cells expressing either mutant ASXL1 or p.Asp868Asn mutant *SETBP1* survived for 6 months after transplantation [60]. Intriguingly, it was noted that *in vivo* the effect of the administration of FTY720, a PP2A activator, was less marked as compared to the efficient repression obtained *in vitro*. These data confirmed the upregulation of the *HOXA9* and *HOXA10* genes and led to the identification of new pathways potentially implicated in disease evolution.

Using RNA-seq data and gene set enrichment analysis (GSEA), several deregulated pathways were identified; among them, attention was focused on the *TGF-β* signaling pathway, in view of its major role in the pathogenesis of AML [65–67] and because some *TGF-β* target genes were reported to be differentially expressed in aCML cells with mutated *SETBP1* [2]. It is known that SKI inhibits TGF-β signaling through interaction with SMAD proteins; as *SETBP1* owns a SKI homologous domain it could be speculated that it has the same regulatory function [68–70]. The down-regulation of *TGF-β* receptors and of *SMAD2/3* targets was observed, all components of the *TGF-β* pathway that had been reported to be altered in MDS [71].

All this evidence shows that the gain of function determined by the mutation of *SETBP1* allows the new protein to interfere with different downstream pathways such as apoptosis, differentiation and self-renewal through alterations of the normal function of the PP2A, *HOXA* genes and *TGF-β* signaling pathway. Thus the combination of *ASXL1* and *SETBP1* mutations activates a stem cell signature and plays a main role in the mechanism of transformation [60] (Figure 2).

These data are important in order to find potential targets for future therapies in high-risk MDS. Furthermore, the effect of mutant SETBP1 on the activity of the *TGF-β* pathway could reveal a link with the SGS phenotype, given the crucial role of this cytokine in bone formation and remodeling [72]. The number of SGS patients described is too small and with limited follow-up, so a predisposition to myeloid neoplasms has not yet been reported and supplementary studies will be required to confirm this hypothesis.

More recently, both the overexpression of wild-type SETBP1 and the presence of a mutant SETBP1 were shown to be capable, alone, of inducing AML in a murine model [73, 74]. Again it was clear that *SETBP1* mutations have a significantly higher oncogenic potential than wild-type *SETBP1*, triggering leukemia with a shorter latency and greater penetrance. At molecular level, a new transcriptional target was found in *MYB*, a transcription factor essential for hematopoiesis [75] that acts as a direct activator of oncogenes such as *MYC* [76], *CCNB1* [77], *BCL2* [76, 78], *SMYD2* [79], and *GFI1* [80], or as a repressor of differentiation regulators such as *SFPI1*, *RUNX1*, *JUNB*, and *CEBPB* [81]. Indeed, *MYB* is, in turn, a target of oncogenes such as *HOXA9* and *MLL* fusions [82], and contributes to a leukemia stem cell maintenance signature [83] conferring a self-renewal capability to myeloid progenitors. *MYB* knockdown experiments provoked differentiation in myeloid progenitors immortalized by both wild-type and mutant *SETBP1*. Intriguingly, both wild type and mutant SETBP1 proteins were found directly bound to *MYB*, in the promoter regions but also introns 2 and 3, suggesting that SETBP1 regulates both transcriptional activation and elongation (Figure 2). As mutant SETBP1 proteins showed a higher transcriptional ability, Nguyen et al. suggested that, besides the increased stability of the protein, mutants could have an enhanced DNA-binding activity and/or that mutations could affect the interaction of SETBP1 with unknown key transcriptional co-factors or repressors [74]. Moreover, a novel function of SETBP1 as a transcriptional repressor through the recruitment of the Nucleosome Remodeling Deacetylase (NuRD) complex was proposed. By means of this ability, SETBP1 could directly repress the transcription of the tumor suppressor gene *RUNX1*, a mechanism that is critical for *SETBP1*-induced transformation [73] (Figure 2).

### *SETBP1* mutations in hematological malignancies: nature, frequency and concomitant alterations

**Figure 3:**
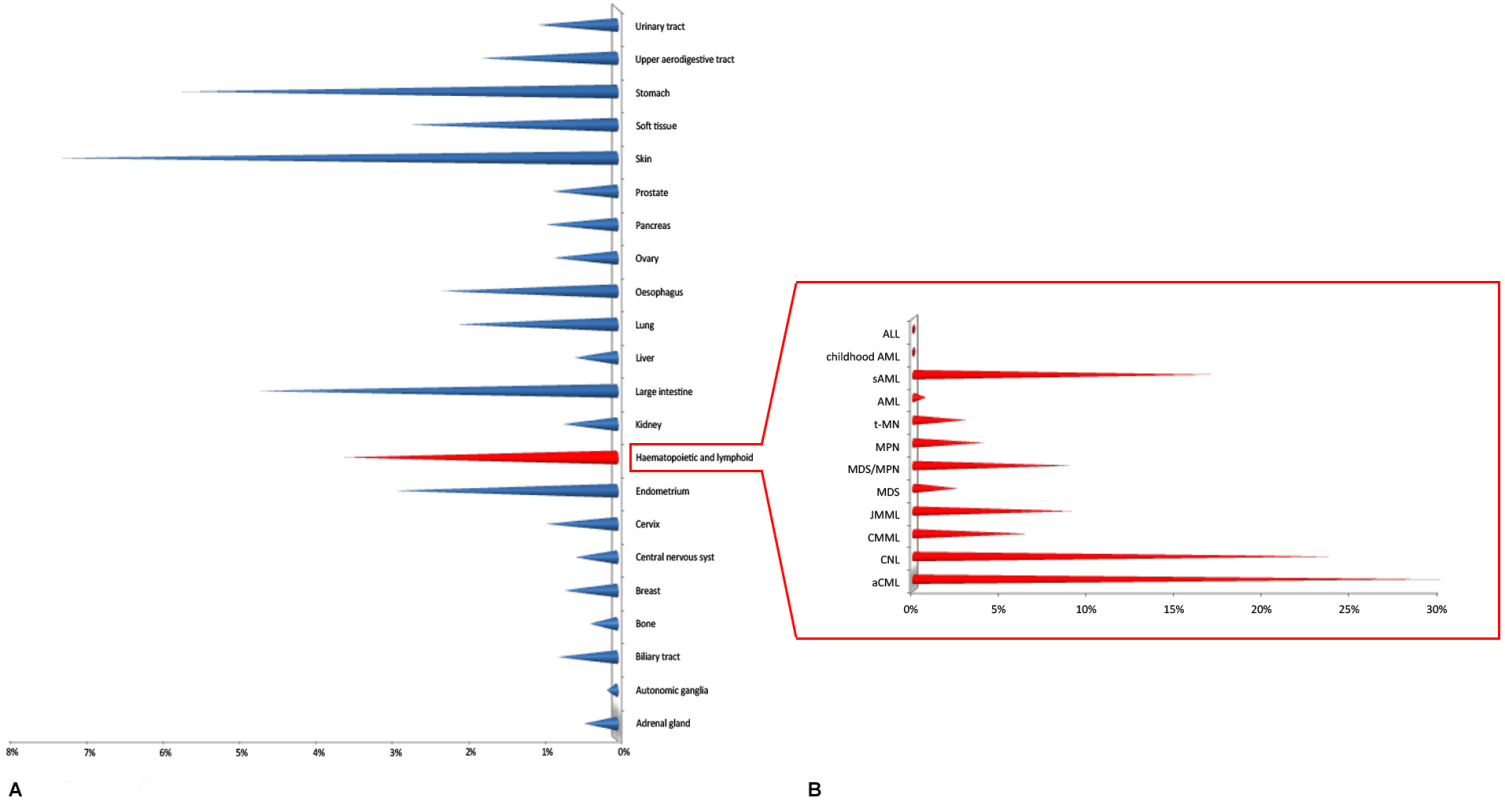
**(A)** Overview of *SETBP1* mutation frequencies in a selection of tumors based on the Cosmic database (Nov 2016). **(B)** SETBP1 mutation frequencies in hematologic neoplasms. Abbreviations: ALL, Acute Lymphoblastic Leukemia; childhood AML, childhood Acute Myeloid Leukemia; sAML, secondary Acute Myeloid Leukemia; AML, Acute Myeloid Leukemia; t-MN, therapy-related Myeloid Neoplasms; MPN, Myeloproliferative Neoplasm; MDS/MPN, Myelodysplastic syndrome/Myeloproliferative neoplasm overlap syndromes; MDS, Myelodysplastic syndrome; JMML, Juvenile Myelomonocytic Leukemia; CMML, Chronic Myelomonocytic Leukemia; CNL, Chronic Neutrophilic Leukemia; aCML, atypic Chronic Myeloid Leukemia.

The discovery of somatic mutation associated with hematological diseases and the advent of Next-Gen sequencing studies paved the way to unveiling many missense mutations within the *SETBP1* gene (Figure 3A). Several studies were focused on analysis of the prevalence, clinical and prognostic value of *SETBP1* mutations in myeloid malignancies other than aCML. In some cases the mutational status was investigated through Sanger sequencing, in others massive or targeted Next-Gen sequencing was applied; often the occurrence of *SETBP1*-mutations was studied in relation to the presence of concomitant mutated genes known to be important in the leukemogenic process.

Reports by several groups confirmed that the *SETBP1* mutation is an important event in various classes of myeloid malignancies including CMML, CNL (Chronic Neutrophilic Leukemia), JMML, MDS, MDS/MPN (Myelodysplastic/Myeloproliferative neoplasms), and AML (Figure 3B).

All reported mutations were missense; when analyzed, the mutational load was almost always 10–50%, representative of a heterozygous status; only few cases showed a homozygous mutation.

The most prevalent mutations were p.Asp868Asn, p.Gly870Ser, p.Ileu871Thr and p.Gly870Asp (38%, 29%, 9%, 3%, respectively – Cosmic release Nov 2016, Figure 4). Sometimes different mutations were reported in the same patient [84].

**Figure 4:**
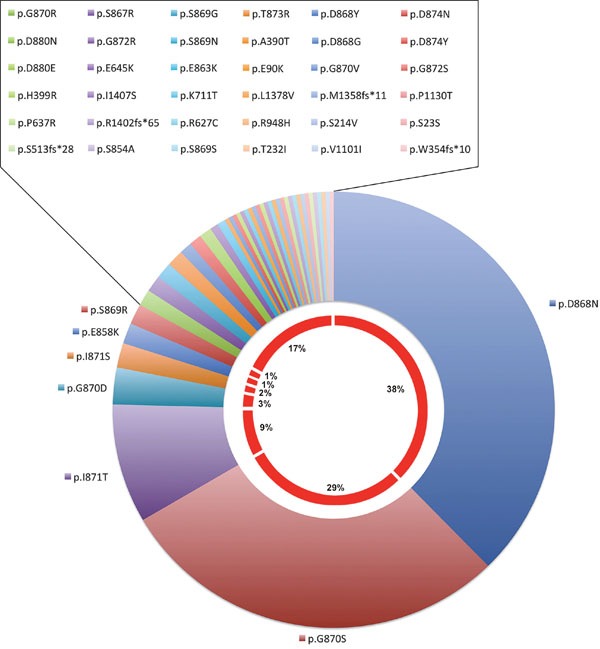
*SETBP1* mutation type frequencies in Haematopoietic and lymphoid neoplasms based on the Cosmic database (release Nov 2016)

Indeed, sorting of early hematopoietic stem cells, multipotent progenitors, common myeloid progenitors, and granulocyte-monocyte progenitors cells clarified which hematopoietic progenitor compartments allow the onset of *SETBP1* mutations. Using droplet digital polymerase chain reaction (ddPCR) analysis, *SETBP1* mutations were detected in all four compartments corroborating the concept that *SETBP1* mutations occur in early cancer-initiating cells [85].

As regards the frequency, *SETBP1* mutations were detected in about 30% of aCML patients [2, 84].

In CNL, frequently *SETBP1* mutations are associated with mutations in the *CSF3R* gene. Several studies reported different frequencies of the *SETBP1* mutation (min 10% – max 38%) [84, 86–88], probably due to different sizes of patient cohorts.

More than 90% of CMML patients show somatic mutations; the most frequently mutated genes are *TET2* (50–60%), *ASXL1* (40–50%), *SRSF2* (40–50%). In this class of myeloid malignancies, *SETBP1* mutations were observed in about 4-7% of patients when the analysis was performed with conventional sequencing methods [84, 89–91]; instead, when deep sequencing was employed, the mutation rate rose to 15%-19% [58, 92]. The most recurrent concomitant mutations were *ASXL1* and *TET2* [93]; *SETBP1* mutations were more frequent in *ASXL1*-mutated CMML patients (67 vs 33%), and less frequent in *TET2*-mutated patients (25 vs 64%) [89].

JMML is a pathology depicted by a very low gene mutation frequency as compared to other neoplasms such as CMML; somatic or germline *RAS* pathway involvement occurs in 89% of cases, and frequently, secondary alterations involve *SETBP1* and *JAK3* genes. About 8-10% of JMML patients showed *SETBP1* mutations [59, 94, 95], even if it is thought that rare subclones below the limits of detection of deep sequencing are present at diagnosis in a large portion of patients who relapse. This hypothesis was demonstrated by Stieglitz et al. using ddPCR, with a limit of detectable events as low as 0.001% [96]; in a cohort of 56 JMML patients they identified *SETBP1* mutations in 17 cases (30%) [85].

In MDS, the *SETBP1* mutation rate is about 2-3%, frequently accompanied by concurrent mutations in other targets such as *ASXL1*, *EZH2* and *SRSF2* [60, 64], and might be associated with distinct cytogenetic aberrations involving chromosomes 7 (-7/del(7q)) and 17 (i(17)(q10)) [2, 58, 84, 89, 97–99]. In particular, *SETBP1* mutations were overrepresented in patients with a sole i(17)(q10) (41-54%) as compared to cases with other cytogenetic rearrangements, and were mutually exclusive with *TP53* mutations [84, 100].

Depending on the sequencing methodology, the mutational detection rate reported in sAML varied between studies; using conventional or massive sequencing the mutational frequencies were 1,7% [89] and 17% [58], respectively; whereas in primary AML the SETBP1 mutation rate was <1% [58]. There was a clear association with *CBL* gene mutations; the *CBL*-mutated clones were found to be significantly smaller than *SETBP1*-mutated clones; this suggested that *CBL* mutations were acquired later than *SETBP1* mutations [58].

Regarding the MDS/MPN category, *SETBP1* mutations might show a frequency of about 9% as compared with only 4% in the MPN category [2, 84]. The most frequently observed concomitant mutations were *ASXL1* and *CBL*; instead, the occurrence of *JAK2* and *TET2* mutations was observed to be mutually exclusive with that of *SETBP1* [84]. *SETBP1* mutations have a causative role in the phenomenon of dysplasia in granulopoiesis and megakaryopoiesis: the bone marrow cytomorphology of *SETBP1* mutated cases presents a characteristic phenotype with an increased dysplastic granulopoiesis and megakaryopoiesis strongly linked to the MDS/MPN category, and in particular to aCML [84].

In therapy-related myeloid neoplasms (t-MN) only 3% of patients presented *SETBP1* mutations [101, 102]. Indeed, *SETBP1* mutations do not seem to be involved in the leukemogenesis of acute lymphoblastic leukemia (ALL) [103] and childhood AML [104].

Analysis of the sequential order of acquisition of *SETBP1* mutations and cytogenetic aberrations as well as mutations in *ASXL1* and *TET2* was performed in 22 MDS/CMML cases in transformation to AML: 15 cases (68.2%) presented mutations in at least one of the three genes during the course of the disease, four cases showed the acquisition of *SETBP1* mutations during leukemic evolution, in one case the *SETBP1* mutation was acquired before harboring the i(17)(q10) marker. However, no clear pattern in the timing of mutation acquisition was observed [98]. In another study, sequential analysis of the *SETBP1* mutation during the clinical course was also performed on 270 samples from 109 patients, among whom 8 patients bore *SETBP1* mutations at diagnosis [64]. In these latter patients the original *SETBP1* mutations were retained, even if the mutant level in one of them was much reduced at the time of AML transformation; on the other hand, 2 of the 101 *SETBP1*-wild type patients acquired novel *SETBP1* mutations during the follow-up. Among the 8 *SETBP1*-mutated patients, 6 presented transformation to acute leukemia and 1 showed disease progression. All these findings suggest that the *SETBP1* mutation could be acquired during the clinical course, implying that it might play a role in disease progression [58], but that perhaps it is not a good biomarker for monitoring the treatment response [64].

### Clinical correlations

Several reports proposed somatic *SETBP1* mutations as a new independent prognostic marker associated with significantly shorter survival and higher white blood cell counts [89, 94].

In aCML, univariate analyses showed no significant differences in terms of age, hemoglobin concentration, platelet counts, and sex distribution. *SETBP1*-mutated cases showed a worse prognosis and higher white blood cell counts at diagnosis [2].

In the case of CNL, the *CSF3R* mutation status did not affect survival, whereas *SETBP1*-mutated patients showed a trend toward refractoriness to treatment and shorter survival, especially when *CSF3R* mutations were co-expressed [86, 105].

In CMML *SETBP1*-mutated patients had a significantly inferior OS and AML-free survival [89, 90, 106]; in multivariate analysis, *SETBP1* mutations maintained the negative prognostic impact [90]. These observations supported the possibility of incorporating *SETBP1* mutations into current prognostic models. Therefore, Elena et al. recently developed a CMML-specific prognostic scoring system (CPSS) based on clinical parameters and cytogenetics that integrated *RUNX1*, *NRAS*, *SETBP1*, and *ASXL1* mutations, defining a clinical/molecular CPSS (CPSS-Mol) model capable of identifying four risk groups with a markedly different median OS and cumulative incidence of leukemic evolution [107]. This study confirmed the prognostic value of *ASXL1* mutations, and highlighted the observation that mutations in *RUNX1*, *NRAS*, and *SETBP1* had an additional independent prognostic value in CPSS cytogenetic risk groups [107].

In JMML, the observation that *SETBP1* mutations occur only in a subpopulation of leukemic cells prompted the view that they may be involved in the evolution rather than at the beginning of leukemia, and are associated with a dismal prognosis. In fact, patients with secondary mutations showed shorter survival than those without mutations; further, patients with JMML who survived without hematopoietic stem cell transplantation did not harbor secondary mutations [85, 94]. Some authors proposed the possibility that the presence of *SETBP1* mutations at subclonal level at diagnosis could be considered as an independent biomarker for poor prognosis that could improve the risk stratification to make an early identification of those patients that should be scheduled for hematopoietic stem cell transplant [85].

In MDS, the presence of the *SETBP1* mutation predicted a poorer OS and higher probability of AML transformation. Indeed, the association of mutations in *SETBP1* with some chromosomes aberrations and *ASXL1*, *EZH2* and *CBL* mutations has been described in MDS and sAML. These genetic markers were associated *per se* with a shorter OS and increased risk of disease progression [58, 108, 109], and the independence of *SETBP1* mutations as a prognostic factor could not always be demonstrated [58, 64]. However, multivariate analysis of OS performed in 64 MDS patients with *ASXL1* mutations showed that the *SETBP1* mutation was an independent poor prognostic factor regardless of age, the 2008 WHO classification and International Prognostic Scoring System (IPSS) classification [60].

Unlike MDS and sAML, *SETBP1* mutations seem not to have a role in the pathogenesis of de novo AML [110], even if an alteration of the *SETBP1* expression levels was found to be associated with a poor prognosis in elderly AML patients [21].

Recently, Shou et al. conducted a meta-analysis to investigate the prognostic effect of SETBP1 in MDS, CMML, and CNL [106]. Through a rigorous selection, a total of 12 key studies with 2321 patients were chosen: 4 studies for MDS, 5 studies for CMML, and 3 studies for CNL. The results confirmed that in MDS and CMML, but not in CNL, SETBP1 mutations are strongly associated with a poorer survival, and that the prognostic impact of SETBP1 mutations is similar to that of ASXL1 mutations [106].

### Response to treatment and promising therapeutic opportunities

It is currently not known whether the presence of *SETBP1* mutations could contribute to the ineffectiveness of therapy. Although *SETBP1* mutations seemed to be associated with primary chemoresistance and induction failure in some AML cases, their prevalence was relatively low [111].

Recently, the plating of cryopreserved samples from serial time-points during follow-up of a JMML relapsed patient demonstrated that the number of cells that were heterozygous or homozygous for the *SETBP1* mutation increased at each time-point despite intensive treatment, suggesting a resistance to traditional cytotoxic therapy [85].

Interestingly, two other studies reported the single cases of one CNL and one aCML, who both co-expressed CSF3R T618I and SETBP1 mutations that proved refractory to ruxolitinib treatment, after failure of hydroxyurea to control progressive neutrophilic leukocytosis [105, 112]. In the case with CNL, *in vitro* studies of the patient's double-mutant myeloid cells demonstrated resistance to the JAK inhibitor treatment [105]. The further observation of a similar case of aCML positive to CSFR3 T618I but wild type for SETBP1 who responded to ruxolitinib, further supported the hypothesis of the role of the *SETBP1* mutation in inducing treatment refractoriness [113]. Despite this, more recently it was reported another CNL patient with mutations in *CSF3R* and *SETBP1*, treated with ruxolitinib, showing clonal evolution with reduction of the *CSF3R* and *SETBP1* mutations allele burden [114]. Therefore, likely due to the paucity of reports, it is still unclear what is the role of the *SETBP1* mutation in relation to response to treatment and to disease evolution.

The association of *SETBP1* activation with poor prognosis in many hematological diseases suggests that the identification of specific therapeutic strategies for these patients may provide an advantage, increasing the cure rate and survivals.

PP2A inactivation is a recurrent event that has been proposed as an important mechanism in the leukemogenic transformation of AML; SETBP1 activation is one of the mechanisms that lead to functional loss of PP2A activity. Pharmacological activation of PP2A seems to offer a future therapeutic alternative as *in vitro* PP2A restoration by PADs (PP2A-activating drugs) reverses some of the leukemogenic features [115, 116].

Likewise, as the *SETBP1* mutation seems to act in repressing the expression of some crucial differentiating genes such as *RUNX1* via the recruitment of a nucleosome remodeling deacetylase, treatment with class I histone deacetylase (HDAC) inhibitors could be a promising strategy to treat human myeloid leukemias with SETBP1 activation [73]. *In vitro* treatment with these inhibitors has been demonstrated to lead to an efficient differentiation of SETBP1 activation-induced leukemia cells, and to significantly extend the survival of mice transplanted with such leukemias [73].

Furthermore, the finding of interplay between *MYB* and mutant or wild type SETBP1 suggests that MYB inhibition could be a promising approach for treating myeloid neoplasms with SETBP1 activation. *In vitro* experiments with primary cultures from cells of a CMML patient with *SETBP1* mutations showed that *MYB* gene knockdown dramatically inhibited colony-forming capability [74]; indeed, it seems that leukemia cells are more sensitive to a reduction of MYB activity than normal hematopoietic progenitors [117, 118] and that interaction of MYB with P300 is required for MYB-mediated leukemia transformation, but is less critical for normal hematopoiesis [119]. In the light of this observation, the triterpenoid Celastrol, a recently identified inhibitor of this interaction, offers a treatment opportunity, as it has been demonstrated *in vitro* and *in vivo* to be efficient in inhibiting the growth of mouse AML cells, while sparing the expansion of normal bone marrow progenitors [120].

## CONCLUDING REMARKS

All the reviewed studies clearly demonstrate a role for *SETBP1* as an oncogenic factor with a double activity, both as a negative regulator of PP2A activity and as transcriptional regulator. However, our knowledge of SETBP1-regulated signaling pathways is still limited. Apart from the regulation of the *HOXA* gene cluster, *RUNX1* and *MYB*, some reports also demonstrated that many TGF-β responsive genes were targeted by SETBP1-mutant proteins. This consideration implies that *SETBP1* may have a role in the regulation of other genes. Conditional and/or tissue-specific induced *SETBP1*-mutated expression may help to identify the crucial pathways that are affected by alterations of *SETBP1* normal function.

It is now evident that the *SETBP1* mutation can be an important factor in cancer development, progression and maybe resistance. Gaining an understanding of the specific cellular functions and related pathways of both the wild-type and mutant SETBP1 proteins will be crucial to identify new targets for therapeutic treatment and so improve outcomes for patients with myeloid malignancies who carry *SETBP1* mutations.

The diverse combinations of mutations detected in some cases of myeloid neoplasms imply a multi-step mechanism of disease pathogenesis. This variable mutational spectrum suggests a complex pathway from driver mutation to clonal evolution to clonal dominance and finally to the onset of the disease. Understanding the role of *SETBP1* mutations in this pathogenic mechanism will help to provide the basis for risk stratification of patients and clinical decision-making.

As *SETBP1* mutations are also seen in other cancer types, like tumors arising in children with SGS, understanding the role of *SETBP1* in hematopoietic neoplasms will contribute to a better understanding of the oncogenic mechanism of other tumors and so to establishing an adequate treatment strategy.

## References

[1] Minakuchi M, Kakazu N, Gorrin-Rivas MJ, Abe T, Copeland TD, Ueda K, Adachi Y (2001). Identification and characterization of SEB, a novel protein that binds to the acute undifferentiated leukemia-associated protein SET. Eur J Biochem.

[2] Piazza R, Valletta S, Winkelmann N, Redaelli S, Spinelli R, Pirola A, Antolini L, Mologni L, Donadoni C, Papaemmanuil E, Schnittger S, Kim DW, Boultwood J (2013). Recurrent SETBP1 mutations in atypical chronic myeloid leukemia. Nat Genet.

[3] Li M, Makkinje A, Damuni Z (1996). The myeloid leukemia-associated protein SET is a potent inhibitor of protein phosphatase 2A. J Biol Chem.

[4] Beresford PJ, Zhang D, Oh DY, Fan Z, Greer EL, Russo ML, Jaju M, Lieberman J (2001). Granzyme A activates an endoplasmic reticulum-associated caspase-independent nuclease to induce single-stranded DNA nicks. J Biol Chem.

[5] Fan Z, Beresford PJ, Oh DY, Zhang D, Lieberman J (2003). Tumor suppressor NM23-H1 is a granzyme A-activated DNase during CTL-mediated apoptosis, and the nucleosome assembly protein SET is its inhibitor. Cell.

[6] Janssens V, Goris J (2001). Protein phosphatase 2A: a highly regulated family of serine/threonine phosphatases implicated in cell growth and signalling. Biochem J.

[7] Wang GL, Iakova P, Wilde M, Awad S, Timchenko NA (2004). Liver tumors escape negative control of proliferation via PI3K/Akt-mediated block of C/EBP alpha growth inhibitory activity. Genes Dev.

[8] Kawabe T, Muslin AJ, Korsmeyer SJ (1997). HOX11 interacts with protein phosphatases PP2A and PP1 and disrupts a G2/M cell-cycle checkpoint. Nature.

[9] Mumby M (2007). PP2A: unveiling a reluctant tumor suppressor. Cell.

[10] Westermarck J, Hahn WC (2008). Multiple pathways regulated by the tumor suppressor PP2A in transformation. Trends Mol Med.

[11] Janssens V, Goris J, Van Hoof C (2005). PP2A: the expected tumor suppressor. Curr Opin Genet Dev.

[12] Schönthal AH (2001). Role of serine/threonine protein phosphatase 2A in cancer. Cancer Lett.

[13] Deheuninck J, Luo K (2009). Ski and SnoN, potent negative regulators of TGF-beta signaling. Cell Res.

[14] Cohen SB, Zheng G, Heyman HC, Stavnezer E (1999). Heterodimers of the SnoN and Ski oncoproteins form preferentially over homodimers and are more potent transforming agents. Nucleic Acids Res.

[15] Oakley K, Han Y, Vishwakarma BA, Chu S, Bhatia R, Gudmundsson KO, Keller J, Chen X, Vasko V, Jenkins NA, Copeland NG, Du Y (2012). Setbp1 promotes the self-renewal of murine myeloid progenitors via activation of Hoxa9 and Hoxa10. Blood.

[16] Bourachot B, Yaniv M, Muchardt C (1999). The activity of mammalian brm/SNF2alpha is dependent on a high-mobility-group protein I/Y-like DNA binding domain. Mol Cell Biol.

[17] Cairns BR, Schlichter A, Erdjument-Bromage H, Tempst P, Kornberg RD, Winston F (1999). Two functionally distinct forms of the RSC nucleosome-remodeling complex, containing essential AT hook, BAH, and bromodomains. Mol Cell.

[18] Xiao H, Sandaltzopoulos R, Wang HM, Hamiche A, Ranallo R, Lee KM, Fu D, Wu C (2001). Dual functions of largest NURF subunit NURF301 in nucleosome sliding and transcription factor interactions. Mol Cell.

[19] Manfredini R, Zini R, Salati S, Siena M, Tenedini E, Tagliafico E, Montanari M, Zanocco-Marani T, Gemelli C, Vignudelli T, Grande A, Fogli M, Rossi L (2005). The kinetic status of hematopoietic stem cell subpopulations underlies a differential expression of genes involved in self-renewal, commitment, and engraftment. Stem Cells.

[20] Jin S, Zhao H, Yi Y, Nakata Y, Kalota A, Gewirtz AM (2010). c-Myb binds MLL through menin in human leukemia cells and is an important driver of MLL-associated leukemogenesis. J Clin Invest.

[21] Cristóbal I, Blanco FJ, Garcia-Orti L, Marcotegui N, Vicente C, Rifon J, Novo FJ, Bandres E, Calasanz MJ, Bernabeu C, Odero MD (2010). SETBP1 overexpression is a novel leukemogenic mechanism that predicts adverse outcome in elderly patients with acute myeloid leukemia. Blood.

[22] Hoischen A, van Bon BW, Gilissen C, Arts P, van Lier B, Steehouwer M, de Vries P, de Reuver R, Wieskamp N, Mortier G, Devriendt K, Amorim MZ, Revencu N (2010). De novo mutations of SETBP1 cause Schinzel-Giedion syndrome. Nat Genet.

[23] Albano F, Anelli L, Zagaria A, Coccaro N, Casieri P, Minervini A, Specchia G (2012). SETBP1 and miR_4319 dysregulation in primary myelofibrosis progression to acute myeloid leukemia. J Hematol Oncol.

[24] Schinzel A, Giedion A (1978). A syndrome of severe midface retraction, multiple skull anomalies, clubfeet, and cardiac and renal malformations in sibs. Am J Med Genet.

[25] Filges I, Shimojima K, Okamoto N, Röthlisberger B, Weber P, Huber AR, Nishizawa T, Datta AN, Miny P, Yamamoto T (2011). Reduced expression by SETBP1 haploinsufficiency causes developmental and expressive language delay indicating a phenotype distinct from Schinzel-Giedion syndrome. J Med Genet.

[26] Schinzel A, Binkert F, Lillington DM, Sands M, Stocks RJ, Lindenbaum RH, Matthews H, Sheridan H (1991). Interstitial deletion of the long arm of chromosome 18, del(18)(q12.2q21.1): a report of three cases of an autosomal deletion with a mild phenotype. J Med Genet.

[27] Hishimura N, Watari M, Ohata H, Fuseya N, Wakiguchi S, Tokutomi T, Okuhara K, Takahashi N, Iizuka S, Yamamoto H, Mishima T, Fujieda S, Kobayashi R (2016). Genetic and prenatal findings in two Japanese patients with Schinzel-Giedion syndrome. Clin Case Rep.

[28] Herenger Y, Stoetzel C, Schaefer E, Scheidecker S, Manière MC, Pelletier V, Alembik Y, Christmann D, Clavert JM, Terzic J, Fischbach M, De Saint Martin A, Dollfus H (2015). Long term follow up of two independent patients with Schinzel-Giedion carrying SETBP1 mutations. Eur J Med Genet.

[29] Takeuchi A, Okamoto N, Fujinaga S, Morita H, Shimizu J, Akiyama T, Ninomiya S, Takanashi J, Kubo T (2015). Progressive brain atrophy in Schinzel-Giedion syndrome with a SETBP1 mutation. Eur J Med Genet.

[30] Volk A, Conboy E, Wical B, Patterson M, Kirmani S (2015). Whole-Exome Sequencing in the Clinic: Lessons from Six Consecutive Cases from the Clinician's Perspective. Mol Syndromol.

[31] Kishimoto K, Kobayashi R, Yonemaru N, Yamamoto H, Tsujioka T, Sano H, Suzuki D, Yasuda K, Suzuki M, Ando A, Tonoki H, Iizuka S, Uetake K, Kobayashi K (2015). Refractory sacrococcygeal germ cell tumor in Schinzel-Giedion syndrome. J Pediatr Hematol Oncol.

[32] Carvalho E, Honjo R, Magalhães M, Yamamoto G, Rocha K, Naslavsky M, Zatz M, Passos-Bueno MR, Kim C, Bertola D (2015). Schinzel-Giedion syndrome in two Brazilian patients: report of a novel mutation in SETBP1 and literature review of the clinical features. Am J Med Genet A.

[33] Ko JM, Lim BC, Kim KJ, Hwang YS, Ryu HW, Lee JH, Kim JS, Chae JH (2013). Distinct neurological features in a patient with Schinzel-Giedion syndrome caused by a recurrent SETBP1 mutation. Childs Nerv Syst.

[34] López-González V, Domingo-Jiménez MR, Burglen L, Ballesta-Martínez MJ, Whalen S, Piñero-Fernández JA, Guillén-Navarro E (2015). [Schinzel-Giedion syndrome: a new mutation in SETBP1]. [Article in Spanish]. An Pediatr (Barc).

[35] Miyake F, Kuroda Y, Naruto T, Ohashi I, Takano K, Kurosawa K (2015). West syndrome in a patient with Schinzel-Giedion syndrome. J Child Neurol.

[36] Suphapeetiporn K, Srichomthong C, Shotelersuk V (2011). SETBP1 mutations in two Thai patients with Schinzel-Giedion syndrome. Clin Genet.

[37] Su AI, Wiltshire T, Batalov S, Lapp H, Ching KA, Block D, Zhang J, Soden R, Hayakawa M, Kreiman G, Cooke MP, Walker JR, Hogenesch JB (2004). A gene atlas of the mouse and human protein-encoding transcriptomes. Proc Natl Acad Sci USA.

[38] Donnai D, Harris R (1979). A further case of a new syndrome including midface retraction, hypertrichosis, and skeletal anomalies. J Med Genet.

[39] al-Gazali LI, Farndon P, Burn J, Flannery DB, Davison C, Mueller RF (1990). The Schinzel-Giedion syndrome. J Med Genet.

[40] Lehman AM, McFadden D, Pugash D, Sangha K, Gibson WT, Patel MS (2008). Schinzel-Giedion syndrome: report of splenopancreatic fusion and proposed diagnostic criteria. Am J Med Genet A.

[41] Elliott AM, Meagher-Villemure K, Oudjhane K, der Kaloustian VM (1996). Schinzel-Giedion syndrome: further delineation of the phenotype. Clin Dysmorphol.

[42] Maclennan AC, Doyle D, Simpson RM (1991). Neurosonography and pathology in the Schinzel-Giedion syndrome. J Med Genet.

[43] Shah AM, Smith MF, Griffiths PD, Quarrell OW (1999). Schinzel-Giedion syndrome: evidence for a neurodegenerative process. Am J Med Genet.

[44] Robin NH, Grace K, DeSouza TG, McDonald-McGinn D, Zackai EH (1993). New finding of Schinzel-Giedion syndrome: a case with a malignant sacrococcygeal teratoma. Am J Med Genet.

[45] Antich J, Manzanares R, Camarasa F, Krauel X, Vila J, Cusi V (1995). Schinzel-Giedion syndrome: report of two sibs. Am J Med Genet.

[46] Sandri A, Manazza AD, Bertin D, Silengo M, Basso ME, Forni M, Madon E (2003). Schinzel-Giedion syndrome with sacrococcygeal teratoma. J Pediatr Hematol Oncol.

[47] Rodríguez JI, Jiménez-Heffernan JA, Leal J (1994). Schinzel-Giedion syndrome: autopsy report and additional clinical manifestations. Am J Med Genet.

[48] McPherson E, Clemens M, Hoffner L, Surti U (1998). Sacral tumors in Schinzel-Giedion syndrome. Am J Med Genet.

[49] Matsumoto F, Tohda A, Shimada K, Okamoto N (2005). Malignant retroperitoneal tumor arising in a multicystic dysplastic kidney of a girl with Schinzel-Giedion syndrome. Int J Urol.

[50] Beschorner R, Wehrmann M, Ernemann U, Bonin M, Horber V, Oehl-Jaschkowitz B, Meyermann R, Dufke A (2007). Extradural ependymal tumor with myxopapillary and ependymoblastic differentiation in a case of Schinzel-Giedion syndrome. Acta Neuropathol.

[51] Marseglia G, Scordo MR, Pescucci C, Nannetti G, Biagini E, Scandurra V, Gerundino F, Magi A, Benelli M, Torricelli F (2012). 372 kb microdeletion in 18q12.3 causing SETBP1 haploinsufficiency associated with mild mental retardation and expressive speech impairment. Eur J Med Genet.

[52] Cody JD, Sebold C, Malik A, Heard P, Carter E, Crandall A, Soileau B, Semrud-Clikeman M, Cody CM, Hardies LJ, Li J, Lancaster J, Fox PT (2007). Recurrent interstitial deletions of proximal 18q: a new syndrome involving expressive speech delay. Am J Med Genet A.

[53] Buysse K, Menten B, Oostra A, Tavernier S, Mortier GR, Speleman F (2008). Delineation of a critical region on chromosome 18 for the del(18)(q12.2q21.1) syndrome. Am J Med Genet A.

[54] Bouquillon S, Andrieux J, Landais E, Duban-Bedu B, Boidein F, Lenne B, Vallée L, Leal T, Doco-Fenzy M, Delobel B (2011). A 5.3Mb deletion in chromosome 18q12.3 as the smallest region of overlap in two patients with expressive speech delay. Eur J Med Genet.

[55] von Lindern M, Breems D, van Baal S, Adriaansen H, Grosveld G (1992). Characterization of the translocation breakpoint sequences of two DEK-CAN fusion genes present in t(6;9) acute myeloid leukemia and a SET-CAN fusion gene found in a case of acute undifferentiated leukemia. Genes Chromosomes Cancer.

[56] Panagopoulos I, Kerndrup G, Carlsen N, Strömbeck B, Isaksson M, Johansson B (2007). Fusion of NUP98 and the SET binding protein 1 (SETBP1) gene in a paediatric acute T cell lymphoblastic leukaemia with t(11;18)(p15;q12). Br J Haematol.

[57] Neviani P, Santhanam R, Trotta R, Notari M, Blaser BW, Liu S, Mao H, Chang JS, Galietta A, Uttam A, Roy DC, Valtieri M, Bruner-Klisovic R (2005). The tumor suppressor PP2A is functionally inactivated in blast crisis CML through the inhibitory activity of the BCR/ABL-regulated SET protein. Cancer Cell.

[58] Makishima H, Yoshida K, Nguyen N, Przychodzen B, Sanada M, Okuno Y, Ng KP, Gudmundsson KO, Vishwakarma BA, Jerez A, Gomez-Segui I, Takahashi M, Shiraishi Y (2013). Somatic SETBP1 mutations in myeloid malignancies. Nat Genet.

[59] Bresolin S, De Filippi P, Vendemini F, D’Alia M, Zecca M, Meyer LH, Danesino C, Locatelli F, Masetti R, Basso G, Te Kronnie G (2016). Mutations of SETBP1 and JAK3 in juvenile myelomonocytic leukemia: a report from the Italian AIEOP study group. Oncotarget.

[60] Inoue D, Kitaura J, Matsui H, Hou HA, Chou WC, Nagamachi A, Kawabata KC, Togami K, Nagase R, Horikawa S, Saika M, Micol JB, Hayashi Y (2015). SETBP1 mutations drive leukemic transformation in ASXL1-mutated MDS. Leukemia.

[61] Ott MG, Schmidt M, Schwarzwaelder K, Stein S, Siler U, Koehl U, Glimm H, Kühlcke K, Schilz A, Kunkel H, Naundorf S, Brinkmann A, Deichmann A (2006). Correction of X-linked chronic granulomatous disease by gene therapy, augmented by insertional activation of MDS1-EVI1, PRDM16 or SETBP1. Nat Med.

[62] Goyama S, Yamamoto G, Shimabe M, Sato T, Ichikawa M, Ogawa S, Chiba S, Kurokawa M (2008). Evi-1 is a critical regulator for hematopoietic stem cells and transformed leukemic cells. Cell Stem Cell.

[63] Junttila MR, Li SP, Westermarck J (2008). Phosphatase-mediated crosstalk between MAPK signaling pathways in the regulation of cell survival. FASEB J.

[64] Hou HA, Kuo YY, Tang JL, Chou WC, Yao M, Lai YJ, Lin CC, Chen CY, Liu CY, Tseng MH, Huang CF, Chiang YC, Lee FY (2014). Clinical implications of the SETBP1 mutation in patients with primary myelodysplastic syndrome and its stability during disease progression. Am J Hematol.

[65] Jakubowiak A, Pouponnot C, Berguido F, Frank R, Mao S, Massague J, Nimer SD (2000). Inhibition of the transforming growth factor beta 1 signaling pathway by the AML1/ETO leukemia-associated fusion protein. J Biol Chem.

[66] Lin HK, Bergmann S, Pandolfi PP (2004). Cytoplasmic PML function in TGF-beta signalling. Nature.

[67] Imai Y, Kurokawa M, Izutsu K, Hangaishi A, Maki K, Ogawa S, Chiba S, Mitani K, Hirai H (2001). Mutations of the Smad4 gene in acute myelogeneous leukemia and their functional implications in leukemogenesis. Oncogene.

[68] Liu X, Sun Y, Weinberg RA, Lodish HF (2001). Ski/Sno and TGF-β signaling. Cytokine Growth Factor Rev.

[69] Suzuki H, Yagi K, Kondo M, Kato M, Miyazono K, Miyazawa K (2004). c-Ski inhibits the TGF-beta signaling pathway through stabilization of inactive Smad complexes on Smad-binding elements. Oncogene.

[70] Wu JW, Krawitz AR, Chai J, Li W, Zhang F, Luo K, Shi Y (2002). Structural mechanism of Smad4 recognition by the nuclear oncoprotein Ski: insights on Ski-mediated repression of TGF-beta signaling. Cell.

[71] Zhou L, Nguyen AN, Sohal D, Ying Ma J, Pahanish P, Gundabolu K, Hayman J, Chubak A, Mo Y, Bhagat TD, Das B, Kapoun AM, Navas TA (2008). Inhibition of the TGF-beta receptor I kinase promotes hematopoiesis in MDS. Blood.

[72] Mundy GR (1991). The effects of TGF-beta on bone. Ciba Found Symp.

[73] Vishwakarma BA, Nguyen N, Makishima H, Hosono N, Gudmundsson KO, Negi V, Oakley K, Han Y, Przychodzen B, Maciejewski JP, Du Y (2016). Runx1 repression by histone deacetylation is critical for Setbp1-induced mouse myeloid leukemia development. Leukemia.

[74] Nguyen N, Vishwakarma BA, Oakley K, Han Y, Przychodzen B, Maciejewski JP, Du Y (2016). Myb expression is critical for myeloid leukemia development induced by Setbp1 activation. Oncotarget.

[75] Mucenski ML, McLain K, Kier AB, Swerdlow SH, Schreiner CM, Miller TA, Pietryga DW, Scott WJ, Potter SS (1991). A functional c-myb gene is required for normal murine fetal hepatic hematopoiesis. Cell.

[76] Wolff L, Schmidt M, Koller R, Haviernik P, Watson R, Bies J, Maciag K (2001). Three genes with different functions in transformation are regulated by c-Myb in myeloid cells. Blood Cells Mol Dis.

[77] Nakata Y, Shetzline S, Sakashita C, Kalota A, Rallapalli R, Rudnick SI, Zhang Y, Emerson SG, Gewirtz AM (2007). c-Myb contributes to G2/M cell cycle transition in human hematopoietic cells by direct regulation of cyclin B1 expression. Mol Cell Biol.

[78] Frampton J, Ramqvist T, Graf T (1996). v-Myb of E26 leukemia virus up-regulates bcl-2 and suppresses apoptosis in myeloid cells. Genes Dev.

[79] Zuber J, Rappaport AR, Luo W, Wang E, Chen C, Vaseva AV, Shi J, Weissmueller S, Fellmann C, Taylor MJ, Weissenboeck M, Graeber TG, Kogan SC (2011). An integrated approach to dissecting oncogene addiction implicates a Myb-coordinated self-renewal program as essential for leukemia maintenance. Genes Dev.

[80] Zhao L, Ye P, Gonda TJ (2014). The MYB proto-oncogene suppresses monocytic differentiation of acute myeloid leukemia cells via transcriptional activation of its target gene GFI1. Oncogene.

[81] Zhao L, Glazov EA, Pattabiraman DR, Al-Owaidi F, Zhang P, Brown MA, Leo PJ, Gonda TJ (2011). Integrated genome-wide chromatin occupancy and expression analyses identify key myeloid pro-differentiation transcription factors repressed by Myb. Nucleic Acids Res.

[82] Hess JL, Bittner CB, Zeisig DT, Bach C, Fuchs U, Borkhardt A, Frampton J, Slany RK (2006). c-Myb is an essential downstream target for homeobox-mediated transformation of hematopoietic cells. Blood.

[83] Somervaille TC, Matheny CJ, Spencer GJ, Iwasaki M, Rinn JL, Witten DM, Chang HY, Shurtleff SA, Downing JR, Cleary ML (2009). Hierarchical maintenance of MLL myeloid leukemia stem cells employs a transcriptional program shared with embryonic rather than adult stem cells. Cell Stem Cell.

[84] Meggendorfer M, Bacher U, Alpermann T, Haferlach C, Kern W, Gambacorti-Passerini C, Haferlach T, Schnittger S (2013). SETBP1 mutations occur in 9% of MDS/MPN and in 4% of MPN cases and are strongly associated with atypical CML, monosomy 7, isochromosome i(17)(q10), ASXL1 and CBL mutations. Leukemia.

[85] Stieglitz E, Troup CB, Gelston LC, Haliburton J, Chow ED, Yu KB, Akutagawa J, Taylor-Weiner AN, Liu YL, Wang YD, Beckman K, Emanuel PD, Braun BS (2015). Subclonal mutations in SETBP1 confer a poor prognosis in juvenile myelomonocytic leukemia. Blood.

[86] Pardanani A, Lasho TL, Laborde RR, Elliott M, Hanson CA, Knudson RA, Ketterling RP, Maxson JE, Tyner JW, Tefferi A (2013). CSF3R T618I is a highly prevalent and specific mutation in chronic neutrophilic leukemia. Leukemia.

[87] Elliott MA, Pardanani A, Hanson CA, Lasho TL, Finke CM, Belachew AA, Tefferi A (2015). ASXL1 mutations are frequent and prognostically detrimental in CSF3R-mutated chronic neutrophilic leukemia. Am J Hematol.

[88] Ouyang Y, Qiao C, Chen Y, Zhang SJ (2017). Clinical significance of CSF3R, SRSF2 and SETBP1 mutations in chronic neutrophilic leukemia and chronic myelomonocytic leukemia. Oncotarget.

[89] Damm F, Itzykson R, Kosmider O, Droin N, Renneville A, Chesnais V, Gelsi-Boyer V, de Botton S, Vey N, Preudhomme C, Clavert A, Delabesse E, Park S (2013). SETBP1 mutations in 658 patients with myelodysplastic syndromes, chronic myelomonocytic leukemia and secondary acute myeloid leukemias. Leukemia.

[90] Laborde RR, Patnaik MM, Lasho TL, Finke CM, Hanson CA, Knudson RA, Ketterling RP, Pardanani A, Tefferi A (2013). SETBP1 mutations in 415 patients with primary myelofibrosis or chronic myelomonocytic leukemia: independent prognostic impact in CMML. Leukemia.

[91] Patnaik MM, Wassie EA, Lasho TL, Hanson CA, Ketterling R, Tefferi A (2015). Blast transformation in chronic myelomonocytic leukemia: risk factors, genetic features, survival, and treatment outcome. Am J Hematol.

[92] Patnaik MM, Lasho TL, Vijayvargiya P, Finke CM, Hanson CA, Ketterling RP, Gangat N, Tefferi A (2016). Prognostic interaction between ASXL1 and TET2 mutations in chronic myelomonocytic leukemia. Blood Cancer J.

[93] Cui Y, Tong H, Du X, Li B, Gale RP, Qin T, Liu J, Xu Z, Zhang Y, Huang G, Jin J, Fang L, Zhang H (2015). Impact of TET2, SRSF2, ASXL1 and SETBP1 mutations on survival of patients with chronic myelomonocytic leukemia. Exp Hematol Oncol.

[94] Sakaguchi H, Okuno Y, Muramatsu H, Yoshida K, Shiraishi Y, Takahashi M, Kon A, Sanada M, Chiba K, Tanaka H, Makishima H, Wang X, Xu Y (2013). Exome sequencing identifies secondary mutations of SETBP1 and JAK3 in juvenile myelomonocytic leukemia. Nat Genet.

[95] Shiba N, Ohki K, Park MJ, Sotomatsu M, Kudo K, Ito E, Sako M, Arakawa H, Hayashi Y (2014). SETBP1 mutations in juvenile myelomonocytic leukaemia and myelodysplastic syndrome but not in paediatric acute myeloid leukaemia. Br J Haematol.

[96] Hindson BJ, Ness KD, Masquelier DA, Belgrader P, Heredia NJ, Makarewicz AJ, Bright IJ, Lucero MY, Hiddessen AL, Legler TC, Kitano TK, Hodel MR, Petersen JF (2011). High-throughput droplet digital PCR system for absolute quantitation of DNA copy number. Anal Chem.

[97] Visconte V, Tabarroki A, Zhang L, Hasrouni E, Gerace C, Frum R, Ai J, Advani AS, Duong HK, Kalaycio M, Saunthararajah Y, Sekeres MA, His ED (2014). Clinicopathologic and molecular characterization of myeloid neoplasms harboring isochromosome 17(q10). Am J Hematol.

[98] Fernandez-Mercado M, Pellagatti A, Di Genua C, Larrayoz MJ, Winkelmann N, Aranaz P, Burns A, Schuh A, Calasanz MJ, Cross NC, Boultwood J (2013). Mutations in SETBP1 are recurrent in myelodysplastic syndromes and often coexist with cytogenetic markers associated with disease progression. Br J Haematol.

[99] Coccaro N, Tota G, Anelli L, Zagaria A, Casieri P, Cellamare A, Minervini A, Minervini CF, Brunetti C, Ricco A, Orsini P, Cumbo C, Specchia G, Albano F (2015). Centromeric fragment of chromosome 7 in atypical chronic myeloid leukemia with the SET binding protein 1 gene mutation. Leuk Lymphoma.

[100] Adema V, Larráyoz MJ, Calasanz MJ, Palomo L, Patiño-García A, Agirre X, Hernández-Rivas JM, Lumbreras E, Buño I, Martinez-Laperche C, Mallo M, García O, Álvarez S (2015). Correlation of myelodysplastic syndromes with i(17)(q10) and TP53 and SETBP1 mutations. Br J Haematol.

[101] Fabiani E, Falconi G, Fianchi L, Criscuolo M, Leone G, Voso MT (2014). SETBP1 mutations in 106 patients with therapy-related myeloid neoplasms. Haematologica.

[102] Fabiani E, Falconi G, Fianchi L, Criscuolo M, Ottone T, Cicconi L, Hohaus S, Sica S, Postorino M, Neri A, Lionetti M, Leone G, Lo-Coco F, Teresa Voso M (2017). Clonal evolution in therapy-related neoplasms. Oncotarget.

[103] Chen M, Yao H, Chen S, Wang Q, Wang Q, Wen L, Xie J, Qin L, Wu L, Qiu H, Cen J (2014). Rare occurrence of SET binding protein 1 mutation in patients with acute lymphoblastic leukemia, mixed phenotype acute leukemia and chronic myeloid leukemia in blast crisis. Leuk Lymphoma.

[104] Choi HW, Kim HR, Baek HJ, Kook H, Cho D, Shin JH, Suh SP, Ryang DW, Shin MG (2015). Alteration of the SETBP1 gene and splicing pathway genes SF3B1, U2AF1, and SRSF2 in childhood acute myeloid leukemia. Ann Lab Med.

[105] Lasho TL, Mims A, Elliott MA, Finke C, Pardanani A, Tefferi A (2014). Chronic neutrophilic leukemia with concurrent CSF3R and SETBP1 mutations: single colony clonality studies, *in vitro* sensitivity to JAK inhibitors and lack of treatment response to ruxolitinib. Leukemia.

[106] Shou LH, Cao D, Dong XH, Fang Q, Wu Y, Zhang Y, Fei JP, Xu BL (2017). Prognostic significance of SETBP1 mutations in myelodysplastic syndromes, chronic myelomonocytic leukemia, and chronic neutrophilic leukemia: A meta-analysis. PLoS One.

[107] Elena C, Gallì A, Such E, Meggendorfer M, Germing U, Rizzo E, Cervera J, Molteni E, Fasan A, Schuler E, Ambaglio I, Lopez-Pavia M, Zibellini S (2016). Integrating clinical features and genetic lesions in the risk assessment of patients with chronic myelomonocytic leukemia. Blood.

[108] Marisavljević D, Rolović Z, Panitić M, Novak A, Djordjević V, Lazarević V, Bosković D, Colović M (2004). [Chromosome 17 abnormalities in patients with primary myelodysplastic syndrome: incidence and biologic significance]. [Article in Serbian]. Srp Arh Celok Lek.

[109] Schanz J, Tüchler H, Solé F, Mallo M, Luño E, Cervera J, Granada I, Hildebrandt B, Slovak ML, Ohyashiki K, Steidl C, Fonatsch C, Pfeilstöcker M (2012). New comprehensive cytogenetic scoring system for primary myelodysplastic syndromes (MDS) and oligoblastic acute myeloid leukemia after MDS derived from an international database merge. J Clin Oncol.

[110] Thol F, Suchanek KJ, Koenecke C, Stadler M, Platzbecker U, Thiede C, Schroeder T, Kobbe G, Kade S, Löffeld P, Banihosseini S, Bug G, Ottmann O (2013). SETBP1 mutation analysis in 944 patients with MDS and AML. Leukemia.

[111] Brown FC, Cifani P, Drill E, He J, Still E, Zhong S, Balasubramanian S, Pavlick D, Yilmazel B, Knapp KM, Alonzo TA, Meshinchi S, Stone RM (2017). Genomics of primary chemoresistance and remission induction failure in paediatric and adult acute myeloid leukaemia. Br J Haematol.

[112] Ammatuna E, Eefting M, van Lom K, Kavelaars FG, Valk PJ, Touw IP (2015). Atypical chronic myeloid leukemia with concomitant CSF3R T618I and SETBP1 mutations unresponsive to the JAK inhibitor ruxolitinib. Ann Hematol.

[113] Dao KH, Solti MB, Maxson JE, Winton EF, Press RD, Druker BJ, Tyner JW (2014). Significant clinical response to JAK1/2 inhibition in a patient with CSF3R-T618I-positive atypical chronic myeloid leukemia. Leuk Res Rep.

[114] Nooruddin Z, Miltgen N, Wei Q, Schowinsky J, Pan Z, Tobin J, Purev E, Gutman JA, Robinson W, Pollyea DA (2017). Changes in allele frequencies of CSF3R and SETBP1 mutations and evidence of clonal evolution in a chronic neutrophilic leukemia patient treated with ruxolitinib. Haematologica.

[115] Cristóbal I, Garcia-Orti L, Cirauqui C, Alonso MM, Calasanz MJ, Odero MD (2011). PP2A impaired activity is a common event in acute myeloid leukemia and its activation by forskolin has a potent anti-leukemic effect. Leukemia.

[116] Ciccone M, Calin GA, Perrotti D (2015). From the Biology of PP2A to the PADs for Therapy of Hematologic Malignancies. Front Oncol.

[117] Calabretta B, Sims RB, Valtieri M, Caracciolo D, Szczylik C, Venturelli D, Ratajczak M, Beran M, Gewirtz AM (1991). Normal and leukemic hematopoietic cells manifest differential sensitivity to inhibitory effects of c-myb antisense oligodeoxynucleotides: an *in vitro* study relevant to bone marrow purging. Proc Natl Acad Sci USA.

[118] Lidonnici MR, Corradini F, Waldron T, Bender TP, Calabretta B (2008). Requirement of c-Myb for p210(BCR/ABL)-dependent transformation of hematopoietic progenitors and leukemogenesis. Blood.

[119] Pattabiraman DR, McGirr C, Shakhbazov K, Barbier V, Krishnan K, Mukhopadhyay P, Hawthorne P, Trezise A, Ding J, Grimmond SM, Papathanasiou P, Alexander WS, Perkins AC (2014). Interaction of c-Myb with p300 is required for the induction of acute myeloid leukemia (AML) by human AML oncogenes. Blood.

[120] Uttarkar S, Dassé E, Coulibaly A, Steinmann S, Jakobs A, Schomburg C, Trentmann A, Jose J, Schlenke P, Berdel WE, Schmidt TJ, Müller-Tidow C, Frampton J, Klempnauer KH (2016). Targeting acute myeloid leukemia with a small molecule inhibitor of the Myb/p300 interaction. Blood.

